# Elevated serum iron level is a predictor of prognosis in ICU patients with acute kidney injury

**DOI:** 10.1186/s12882-020-01965-9

**Published:** 2020-07-25

**Authors:** Jie Shu, Yufeng Hu, Xueshu Yu, Jiaxiu Chen, Wenwei Xu, Jingye Pan

**Affiliations:** Department of Intensive Care Unit, Wenzhou Medical University, Wenzhou, 325000 Zhejiang, People’s Republic of China

**Keywords:** Acute kidney injury, Iron, Predictor, Mortality

## Abstract

**Background:**

Accumulation of iron is associated with oxidative stress, inflammation, and regulated cell death processes that contribute to the development of acute kidney injury (AKI). We aimed to investigate the association between serum iron levels and prognosis in intensive care unit (ICU) patients with AKI.

**Methods:**

A total of 483 patients with AKI defined as per the Kidney Disease: Improving Global Guidelines were included in this retrospective study. The data was extracted from the single-centre Medical Information Mart for Intensive Care III database. AKI patients with serum iron parameters measured upon ICU admission were included and divided into two groups (low group and high group). The prognostic value of serum iron was analysed using univariate and multivariate Cox regression analysis.

**Results:**

The optimal cut-off value for serum iron was calculated to be 60 μg/dl. Univariable Cox regression analysis showed that serum iron levels were significantly correlated with prognosis of AKI patients. After adjusting for possible confounding variables, serum iron levels higher than 60 μg/dl were associated with increases in 28-day (hazard [HR] 1.832; *P* <  0.001) and 90-day (HR 1.741; *P* <  0.001) mortality, as per multivariable Cox regression analysis.

**Conclusions:**

High serum iron levels were associated with increased short- and long-term mortality in ICU patients with AKI. Serum iron levels measured upon admission may be used for predicting prognosis in AKI patients.

## Background

Acute kidney injury (AKI) is a severe syndrome with high morbidity and significant mortality risk. It is a common complication in patients admitted to hospitals (10–15% of all hospitalizations) [1] and intensive care units (over 50%) [2]. Increasing AKI severity is associated with a stepwise increase in mortality [2]. Given that no targeted therapy can reliably prevent or treat AKI, there is a necessity to choose appropriate biological indicators to predict the prognosis of AKI and enable adequate prevention and treatment.

Iron plays an essential role in many critical cellular functions such as hypoxia signalling, mitochondrial function, erythropoiesis, cell cycle progression, DNA synthesis and repair, and regulation of inflammation. Nevertheless, excess iron is toxic to cells and tissues, including the kidney, due to its ability to cause oxidative stress (OS) and mitochondrial dysfunction, promote inflammation, and regulate cell death [3].

OS, inflammation, and regulated cell death mechanisms are thought to have important roles in AKI [3]. Persistent OS, mitochondrial dysfunction, and inflammation are thought to promote the development of AKI to chronic kidney disease (CKD) [3]. The relationship between iron-related parameters and AKI has been confirmed in several preclinical trials, but few clinical trials researching the prognosis of AKI are available. Therefore, we hypothesized that elevated serum iron is positively associated with poor prognosis in patients with AKI. We thus, investigated the prognostic role of serum iron in short- and long-term mortality of ICU patients with AKI.

## Methods

### Data sources

The data in our study was acquired from a large, single-centre database named Medical Information Mart for Intensive Care (MIMIC-III). It integrates the clinical information of 53,423 patients (aged 16 years or above) who were admitted to critical care units of the Beth Israel Deaconess Medical Centre in Boston, Massachusetts, from 2001 to 2012 [4]. Clinical information collected were coded data, interventions, demographic detail, laboratory test results, medications, and survival data.

### Study patients

A total of 483 adult patients with AKI were included in the final analysis after excluding patients with missing serum iron measurements and repetitive admissions. The occurrence of AKI was defined by Kidney Disease: Improving Global Outcomes (KDIGO) when serum creatinine increased by greater than 50% from baseline or showed 0.3 mg/dL or more increase within 48 h. Patients with AKI were divided into three stages as per KDIGO. Stage 1 patients were those whose creatinine increased by more than 1.5 times baseline or showed 0.3 mg/dL or more increase within any 48 h period, or whose urine volume was less than 0.5 ml/kg for 6–12 h. Stage 2 patients were those whose creatinine increased by more than 2.0 times baseline or whose urine volume was less than 0.5 ml/kg for more than 12 h. Stage 3 patients were those whose creatinine increased by more than 3.0 times baseline or increased to more than 4.0 mg/dl, who required acute dialysis, or whose urine volume was less than 0.3 ml/kg for more than 24 h.

Iron-related parameters measured on the first day after ICU admission such as serum iron, ferritin, and transferrin were acquired. For some patients from whom iron-related parameters were repeatedly measured, the mean value was used. Other information including age (categorized into 16–59 and 60 years or above), gender, comorbidities, stage of AKI, related treatment, other laboratory measurements, and survival data were extracted from the database. All the above data were extracted using structure query language (SQL).

### Outcomes

The primary outcome was the association between serum iron levels and 28-day and 90-day mortality of ICU patients with AKI. The secondary outcomes included the roles of ferritin and transferrin in the prognosis of ICU patients with AKI.

### Statistical analyses

We used the random forest model to impute missing data (Fig. S1 (see Additional file 1)) [5] and analysed the original and imputed data, respectively. Shapiro-Wilk tests were performed and density maps were drawn to determine the normality of the distribution of the continuous variables. Normally distributed continuous variables are reported as mean ± standard deviation (SD), while skewed variables are expressed as medians and interquartile ranges (IQRs). AKI patients were stratified according to receiver operating characteristic (ROC) curve analysis (Fig. S2 (see Additional file 2)). The optimal cut-off value for serum iron was calculated to be 60 μg/dl; thus, patients were divided into two groups (Low group: < 60 μg/dl; High group: ≥60 μg/dl) (more detail in Additional file 2). Boxplots were generated to show the correlation between serum iron levels and different types of survival status. Differences between the death group and survival group were assessed using a Wilcoxon test or t-test. We used log-rank tests to compare the 28-day and 90-day survival rates among different groups and expressed the results as Kaplan–Meier curves. Correlations between iron-related parameters and short- and long-term mortality were determined by the univariate Cox proportional hazards model. The variables analysed in univariate Cox proportional hazards model were selected based on clinical relevance. These variables were finally incorporated into multivariate Cox proportional hazards models to determine the independent effects of serum iron levels on short- and long-term mortality. The hazard ratio (HR) and its 95% confidence interval (CI) were calculated. All comparisons were two-tailed, with *P* <  0.05 considered significant. All statistical analyses were performed using R 3.5.1.

We conducted a sensitivity analyses with the cohort with missing data to further validate the primary outcomes.

## Results

A total of 483 patients with AKI (246 males and 237 females) who fulfilled the KDIGO criteria were included in the final analysis. The demographics, laboratory measurements, stage of AKI, related treatment, and comorbidities are shown in Table 1 (details on baseline characteristics of the original study cohort and baseline characteristics of the patients in two groups after interpolation can be found in the electronic supplementary material, Tables S1 and S2 (see Additional file 3)). More than half the patients were older than 60 years. The majority of patients had Stage 3 AKI. Most patients had a sequential organ failure assessment (SOFA) score greater than 2 points. There were 311 patients in the low group and 172 patients in the high group. The values of transferrin and ferritin were 160.9 ± 58.41 mg/dl and 496.27 ± 392.81 ng/ml, respectively.

**Table 1:** Baseline characteristics of the interpolated study cohort

Characteristics	Values
**Total**	483
**Sex,*n*(%)**	
Female	237 (49.1)
Male	246 (50.9)
**Age, n (%) (years)**	
16–59	196 (40.6)
≥ 60	287 (59.4)
**Comorbidity, n (%)**	
Congestive heart failure	198 (41.0)
Hypertension	255 (52.8)
**SOFA score, n (%)**	
< 2	39 (8.1)
≥ 2	444 (91.9)
**Stage of AKI, n (%)**	
Stage 1	114 (23.6)
Stage 2	115 (23.8)
Stage 3	254 (52.6)
**RRT, n (%)**	123 (25.5)
**Iron group, n (%)**	
Low iron group	311 (64.4)
High iron group	172 (35.6)
**Laboratory measurements**	
Creatinine, mean (SD) (mg/dl)	2.25 (1.59)
Transferrin, mean (SD) (mg/dl)	160.90 (58.41)
Ferritin, mean (SD) (ng/ml)	496.27 (392.81)
**28-day survival status, n (%)**	
Non-death	334 (69.2)
Death	149 (30.8)
**28-day survival times, mean (SD)**	22.34 (9.52)
**90-day survival status, n (%)**	
Non-death	292 (60.5)
Death	191 (39.5)
**90-day survival times, mean (SD)**	61.76 (37.19)

Values are presented as number (%) and mean (SD). *SOFA Score* Sequential Organ Failure Assessment score, *RRT* Renal Replacement Therapy, *SD* standard deviation

**Fig. 1:**
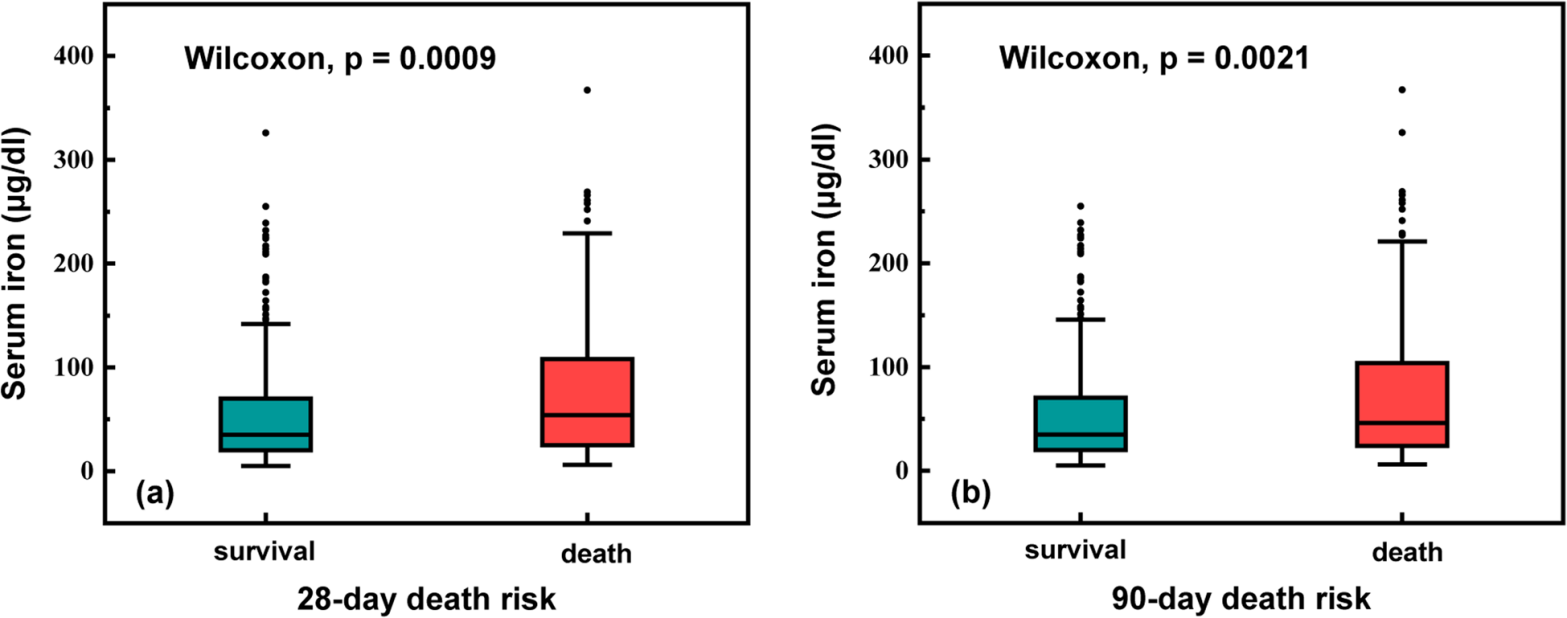
Boxplots exploring the relationship between serum iron levels and different survival status. Serum iron levels were significantly higher in patients with death

**Fig. 2:**
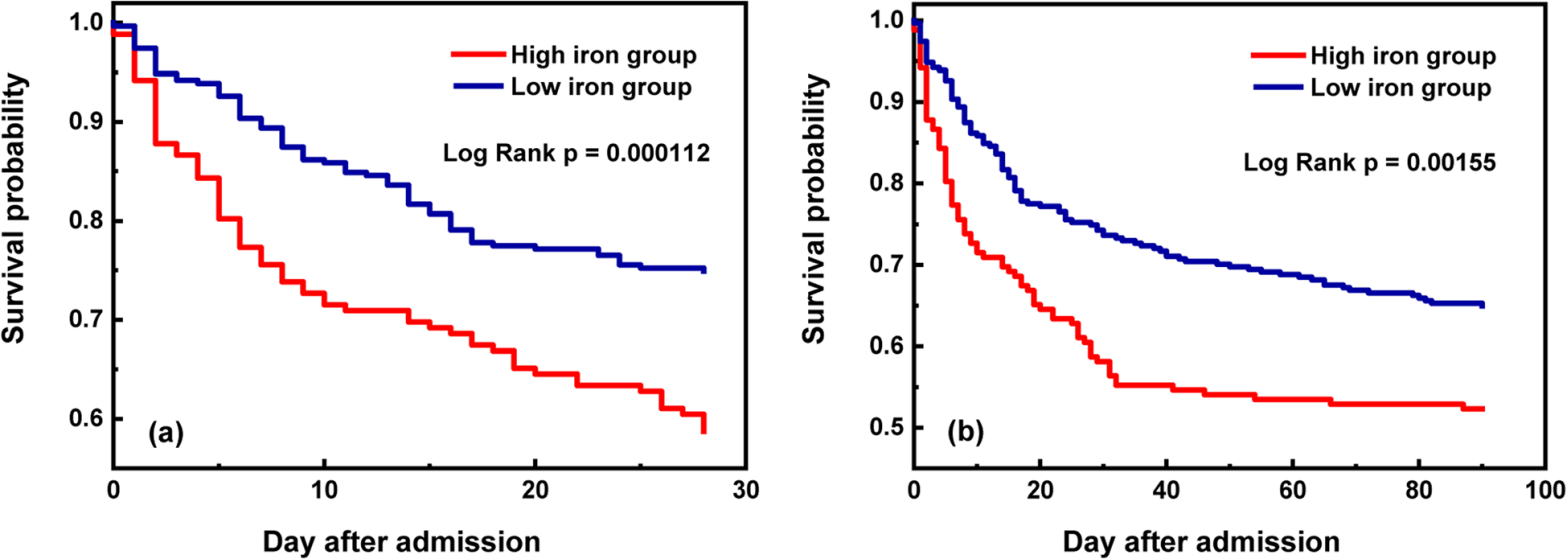
Kaplan-Meier curves demonstrating the association of different serum iron levels and short- and long-term mortality. The short- and long-term mortality increased as serum iron levels increased

The total 28-day and 90-day mortality rates were 30.8 and 39.5%, respectively (Table 2). As shown in the boxplots, significant associations were observed between the 28-day and 90-day survival status values and serum iron levels (*P* <  0.001 and *P* = 0.0021, respectively) (Fig. 1). The 28-day and 90-day mortality risks increased as serum iron levels increased (*P* <  0.001 and *P* = 0.0015, respectively) (Fig. 2). After adjusting for possible confounding variables, significant correlations remained between high serum iron level and 28-day and 90-day mortality risks (HR = 1.832, 95% CI: 1.305–2.573, *P* <  0.001; HR = 1.741, 95% CI: 1.285–2.358, *P* <  0.001, respectively) as per the multivariate Cox proportional hazards models (Tables 3 and 4). We also analysed the original cohort with missing data, and found similar result: 28-day and 90-day mortality increased in patients with high serum iron (Tables S3 and S4 (see Additional file 3)).With respect to the other iron-related measurements, transferrin was associated with increased survival rates in ICU patients with AKI (HR = 0.996, 95% CI: 0.993–0.999, *P* = 0.021; HR = 0.0994, 95% CI: 0.991–0.997, *P* <  0.001, respectively), while ferritin had no significant correlation with 28-day and 90-day mortality risks (HR = 1.001, 95% CI: 1.000–1.001, *P* <  0.05; HR = 1.000, 95% CI: 1.000–1.001, *P* > 0.05, respectively) (Tables 3 and 4).

**Table 2:** Survival outcomes of patients in different serum iron levels groups

Outcomes	Total (***n*** = 483)	Low iron group (***n*** = 311)	High iron group (***n*** = 172)	***P*** Value
28-day mortality, n (%)	149 (30.8)	78 (25.1)	71 (41.3)	< 0.001
90-day mortality, n (%)	191 (39.5)	109 (35.0)	82 (47.7)	0.009

**Table 3:** Cox proportional hazards models exploring the relationship between serum iron levels and 28-day mortality

Factors	Univariate model		Multivariate model	
	HR (95% CI)	***P*** Value	HR (95% CI)	***P*** Value
**Sex (M)**	1.436 (1.037–1.990)	0.029	1.208 (0.760–5.971)	0.263
**Age (years)**				
16–59	Reference	–	Reference	–
≥ 60	1.217 (0.871–1.699)	0.250	1.720 (0.868–1.680)	0.004
**Congestive heart failure**	0.823 (0.591–1.146)	0.248	0.913 (0.633–1.316)	0.624
**Hypertension**	0.759 (0.550–1.047)	0.093	0.717 (0.510–1.010)	0.057
**SOFA score**				
< 2	Reference	–	Reference	–
≥ 2	3.653 (1.353–9.866)	0.011	2.130 (0.760–5.971)	0.151
**Stage of AKI**				
Stage 1	Reference	–	Reference	–
Stage 2	1.231 (0.709–2.136)	0.461	1.152 (0.661–2.006)	0.618
Stage 3	2.131 (1.353–3.356)	0.001	1.543 (0.919–2.589)	0.101
**Treatment**				
Non-RRT	Reference	–	Reference	–
RRT	1.663 (1.186–2.334)	0.003	1.008 (0.653–1.556)	0.972
**Creatinine**	1.104 (1.005–1.213)	0.040	0.992 (0.882–1.116)	0.893
**Transferrin**	0.993 (0.990–0.996)	< 0.001	0.996 (0.993–0.999)	0.021
**Ferritin**	1.001 (1.001–1.001)	< 0.001	1.001 (1.000–1.001)	0.029
**Iron group**				
Low iron group	Reference	–		–
High iron group	1.868 (1.354–2.576)	< 0.001	1.832 (1.305–2.573)	< 0.001

*HR* Hazard Ratio, *CI* Confidence Interval, *SOFA* Sequential Organ Failure Assessment, *AKI* Acute Kidney Injury, *RRT* Renal Replacement Therapy

**Table 4:** Cox proportional hazards models exploring the relationship between serum iron levels and 90-day mortality

Factors	Univariate model		Multivariate model	
	HR (95% CI)	***P*** Value	HR (95% CI)	***P*** Value
**Sex (M)**	1.524 (1.142–2.034)	0.004	1.308 (0.976–1.753)	0.072
**Age (years)**				
16–59	Reference	–	Reference	–
≥ 60	1.44 (1.065–1.947)	0.018	1.910 (1.372–2.661)	< 0.001
**Congestive heart failure**	1.012 (0.759–1.348)	0.936	1.115 (0.812–1.531)	0.502
**Hypertension**	0.7821 (0.589–1.039)	0.090	0.703 (0.520–0.951)	0.022
**SOFA score**				
< 2	Reference	–	Reference	–
≥ 2	2.760 (1.297–5.872)	0.008	1.534 (0.694–3.387)	0.290
**Stage of AKI**				
Stage 1	Reference	–	Reference	–
Stage 2	1.119 (0.693–1.807)	0.647	1.102 (0.680–1.786)	0.693
Stage 3	2.030 (1.376–2.995)	< 0.001	1.587 (1.017–2.477)	0.042
**Treatment**				
Non-RRT	Reference	–	Reference	–
RRT	1.505 (1.108–2.043)	0.009	0.816 (0.553–1.203)	0.304
**Creatinine**	1.112 (1.023–1.208)	0.0127	1.030 (0.928–1.143)	0.577
**Transferrin**	0.992 (0.989–0.995)	< 0.001	0.994 (0.991–0.997)	< 0.001
**Ferritin**	1.001 (1.001–1.001)	< 0.001	1.000 (1.000–1.001)	0.086
**Iron group**				
Low iron group	Reference	–	Reference	–
High iron group	1.585 (1.190–2.112)	0.002	1.741 (1.285–2.358)	< 0.001

*HR* Hazard Ratio, *CI* Confidence Interval, *SOFA* Sequential Organ Failure Assessment; *AKI* Acute Kidney Injury, *RRT* Renal Replacement Therapy

## Discussion

In this retrospective study, we investigated the association between high serum iron levels and increased short- and long-term mortality in ICU patients with AKI. Additional analysis showed that transferrin was associated with increased survival rates of ICU patients with AKI.

In the biomedical field, iron, an element essential for life, is a double-edged sword. Iron levels in the body are tightly controlled by cells and systems through the maintenance of a pool of iron available for biosynthetic purposes [6–8]. Plasma transferrin molecules safely carry the ferric state (Fe^3+^) and supply it to cells associated with the abovementioned pool. Serum iron, i.e. transferrin bound iron (TBI), is the physiological source of circulating iron captured by cells on demand. Serum iron is delivered into cells and the metal is released into the cytosol as labile cell iron (LCI) [9]. The normal range of plasma transferrin saturation (TSAT) is usually 20–40% and rarely exceeds 60%, except in systemic iron overload conditions such as in hereditary haemochromatosis [10]. In systemic iron overload, excess iron cannot be matched by sufficient unsaturated iron binding capacity and results in the formation of non-transferrin bound iron (NTBI). A portion of circulating NTBI is readily available to participate in redox cycling and is defined as labile plasma iron (LPI) [11–13]. LPI can infiltrate cells, raise LCI and cause cellular iron overload. Thus, during systemic iron overload, an increase in serum iron may be followed by an increase in LPI when unsaturated iron binding capacity decreases. The potential mechanisms that elevate serum iron levels are significantly associated with mortality in severe AKI patients and may be related to LPI.

Catalytic iron, also known as LPI, is a transitional pool of NTBI. It readily participates in redox cycling and causes damage to cell membranes, proteins, and DNA through redox reaction such as the Fenton reaction [9, 13, 14]. The role of catalytic iron as a critical player in different types of AKI has been demonstrated in many animal models [15, 16]. A study in a rat model of ischaemia/reperfusion injury (IRI) reported no significant changes in total iron, non-haem or ferritin iron levels, but a significant increase in catalytic iron level after reperfusion [17]. IRI models may possess possible self-protection mechanisms for regulating iron homeostasis [18].

In a rat model of cisplatin-induced nephrotoxicity, a key role of iron in mediating tissue damage through hydroxyl radicals (or similar oxidants) was demonstrated [19]. Another study reported the protective effects of hydroxyl radical scavengers and iron chelators on penicillin-induced acute renal failure [20]. Moreover, the protective effect of iron chelator deferoxamine on renal function was identified in rat models [21]. Ikeda Y, et al. showed that restricting dietary iron could inhibit oxidative stress and inflammatory changes, thereby reducing renal tubular interstitial damage [22].

Recent years have seen research on iron-related measurements in humans. Several studies have shown that elevated levels of catalytic iron are associated with increased incidence of AKI triggered by different conditions [13, 23–26]. Hepcidin is an essential regulator of iron homeostasis; it reduces extracellular iron levels by downregulating iron absorption in the duodenum and ferroportin expression and cellular iron release in macrophages [25, 27]. The protective role of hepcidin in AKI provides evidence of the key role of iron in mediating AKI [28]. A study involving 807 patients showed that plasma catalytic iron and hepcidin are possibly useful prognostic indicators for AKI patients [16]. At present, most studies focus on the relationships between iron-related measurements and morbidity of AKI rather than mortality. Few studies have reported the roles of iron-related measurements in mortality of AKI in humans. Our study found that high serum iron levels were significantly associated with short- and long-term mortality of patients with AKI. Clinically, serum iron levels measured upon admission may be used as prognostic markers for AKI, thereby enabling timely interventions aimed at reducing mortality.

In addition, our study found that transferrin was associated with increased survival rates of patients with AKI. Transferrin is an iron-binding blood plasma glycoprotein that controls the level of free iron in body fluids. Recently, it has been shown to be a good indicator of organ failure; elevated transferrin levels have been associated with a reduction in the short-time mortality of patients with decompensated cirrhosis [29] and low serum transferrin levels have been associated with an increased risk in patients with sepsis [30]. As the main protein that binds and transports iron into the circulatory system, transferrin may decrease iron-mediated cell injury by increasingly combining with overloaded iron. The potential mechanisms behind this require further investigation.

Ferritin, an iron binding protein whose function is to store iron in tissue, has the ability to sequester large amounts of intracellular iron and thereby prevent oxidative cellular damage [31]. In humans, there are conflicting reports on the role of ferritin in AKI. Several studies have demonstrated the protective effect of ferritin heavy chain on renal function [32, 33]. Some studies have shown that lower levels of ferritin are associated with increased morbidity of AKI after cardiopulmonary bypass [33, 34]. Dimitrijevic ZM, et al. reported that elevated serum ferritin levels favour renal function recovery [15], but this association was not observed in another study of 120 patients [35]. The latter observation was consistent with our study, in which ferritin had no significant correlation with mortality in ICU patients with AKI. Disturbances in cellular and systemic iron balance and AKI may affect each other as the kidney is an important player in preventing iron loss from the body by reabsorption [3]. Different tubular segments play different roles in handling iron; the proximal tubule has the maximum reabsorption capacity [36, 37]. The kidney reabsorbs iron, even when systemic iron levels are high [3]. Studies have shown that levels of catalytic iron in urine increased, rather than decreased, in AKI patients [38–40], but body iron stores are not low in AKI patients [26, 41]. The iron-mediated mechanisms underlying AKI are complex and may include multiple pathways. Excess iron is associated with OS, and production of oxygen free radicals causes damage to lipids, DNA, and proteins [9]; renal tubular epithelial cells are particularly vulnerable to OS due to the high number of mitochondria in these cells [42]. In a rat model of acute ischaemia, mitochondrial dysfunction caused by OS was seen to lead to the production of proinflammatory cytokines [3]. Free iron can amplify the inflammatory response through the intracellular uptake and catabolism of damaged stored red blood cells via the monocyte-macrophage system [43]. This is amplified by the fact that the inflammatory response is important in the pathogenesis of AKI [3, 44]. Iron-mediated OS, mitochondrial dysfunction, and inflammatory responses are possible mechanisms of AKI. Ferroptosis has also been considered a central player in AKI, characterized by the accumulation of lethal lipid ROS produced by iron-mediated lipid peroxidation [25, 45, 46]. The excess iron in AKI may arise from degraded red blood cells, iron release from ferritin, and from mitochondria rich in haem and non-haem iron [47].

In terms of iron-targeted therapy for AKI, the therapeutic effects of hepcidin, deferoxamine, apolipoprotein, and pharmacologic therapy with apotransferrin and hydroxyl radical scavengers have been reported in animal models [47, 48]. These results, in combination with those from our study, warrant further studies on iron-targeted therapy in patients with AKI.

Biochemical assessments of iron status iron-related indicators, such as serum iron, ferritin, transferrin and TAST have been conducted in several studies. However, the focus of the indicators has mainly been on deficiency states and measurements of iron repletion and overload have received little attention. The measurement of iron-related indicators is generally carried out on fully-automated clinical analysers. Serum iron is measured on chemical analysers through using a colorimetric reaction with ferrine or ferrozine as a chromogen to form a colour complex with iron, while protein-based indicators such as ferritin and transferrin are measured using immunoassays [49]. Whereas the abovementioned laboratory methods are widely available and provide good precision, the comparability of these results across different assay platforms still needs improvement. In addition, the serum iron levels in normal people exhibit diurnal variation, with higher values in the morning than in the afternoon [50]. The above factors may affect the accuracy of iron status measurements.

Our study has several limitations. First, this is a retrospective study with confounding bias due to missing values in the database and some indicators not being recorded in the MIMIC-III database. Second, MIMIC-III is a single-centre database with data between 2001 to 2012; therefore, even though the sample size of our study is large, the information may be relatively old. Third, we selected only one measurement of iron-related parameters on ICU admission as a research indicator and did not monitor the dynamic trend of serum iron level changes. Fourth, C-reactive protein (CRP) as a marker of inflammation is also important for the interpretation of various measured parameters. However, the MIMIC-III database lacks CRP data. In addition, serum iron levels might be influenced by haemolysis, intravenous iron therapy, blood transfusion and muscle damage. We were unable to obtain relevant data to adjust for these confounding effects, which might impact our results.

## Conclusions

We clarified the relationship between elevated serum iron level and increased mortality in ICU patients with AKI and also investigated the protective effect of transferrin on prognosis of AKI. Serum iron levels can indicate the severity and prognosis of AKI and guide clinical decision making and monitoring of disease progression. Further clinical studies on iron-targeted therapy in AKI are required to better understand this process.

## Supplementary information

**Additional file 1: Figure S1.** Pattern of missing data in variables of interest.

**Additional file 2: Figure S2.** Receiver-operating characteristics (ROC) curve analysis of the best cut-off of serum iron.

**Additional file 3: Table S1.** Baseline characteristics of the original study cohort. Values are number (%) and mean (SD). SOFA Score, Sequential Organ Failure Assessment score; RRT, Renal replacement therapy; SD, standard deviation. **Table S2** Baseline characteristics of the patients in two groups after interpolation. Values are number (%) and mean (SD). SOFA Score, Sequential Organ Failure. Assessment score; RRT, Renal replacement therapy; SD, standard deviation. **Table S3.** Cox proportional hazards models exploring the relationship between serum iron levels and 28-day mortality. **Table S4.** Cox proportional hazards models exploring the relationship between serum iron levels and 90-day mortality.

## Data Availability

The datasets used and/or analysed during the current study are available from the corresponding author upon reasonable request.

## Abbreviations

AKI: Acute kidney injury
CI: Confidence interval
CKD: Chronic kidney disease
CRP: C-reactive protein
HR: Hazard ratio
ICU: Intensive Care Unit
IQR: Interquartile range
IRI: Ischaemia/reperfusion injury
KIDGO: Kidney Disease: Improving Global Guidelines
OS: Oxidative stress
ROC: Receiver operating characteristic
RRT: Renal replacement therapy
SD: Standard deviation
SQL: Structure query language
TSAT: Transferrin saturation
