# Supplementary material for: Elevated serum iron level is a predictor of prognosis in ICU patients with acute kidney injury
Source: BMC Nephrol. 2020 Jul 25;21:303. doi: 10.1186/s12882-020-01965-9 (PMC7382811; doi:10.1186/s12882-020-01965-9)

Title： Elevated serum iron level is a predictor of prognosis in ICU patients with acute kidney injury

Jie Shu[1], Yufeng HU[1], Xueshu Yu[1], Jiaxiu Chen[1], Wenwei Xu[1], Jingye Pan[1, *]

Affiliations

[1] Department of Intensive Care Unit, Wenzhou Medical University, Wenzhou, 325000, Zhejiang, People's Republic of China

[*] **Corresponding author:** Jing-Ye Pan, The First Affiliated Hospital of Wenzhou Medical University, Wenzhou, Zhejiang Province, 325000, China. E-mail: wmupanjingye@126.com.

Figure S1. Pattern of missing data in variables of interest.

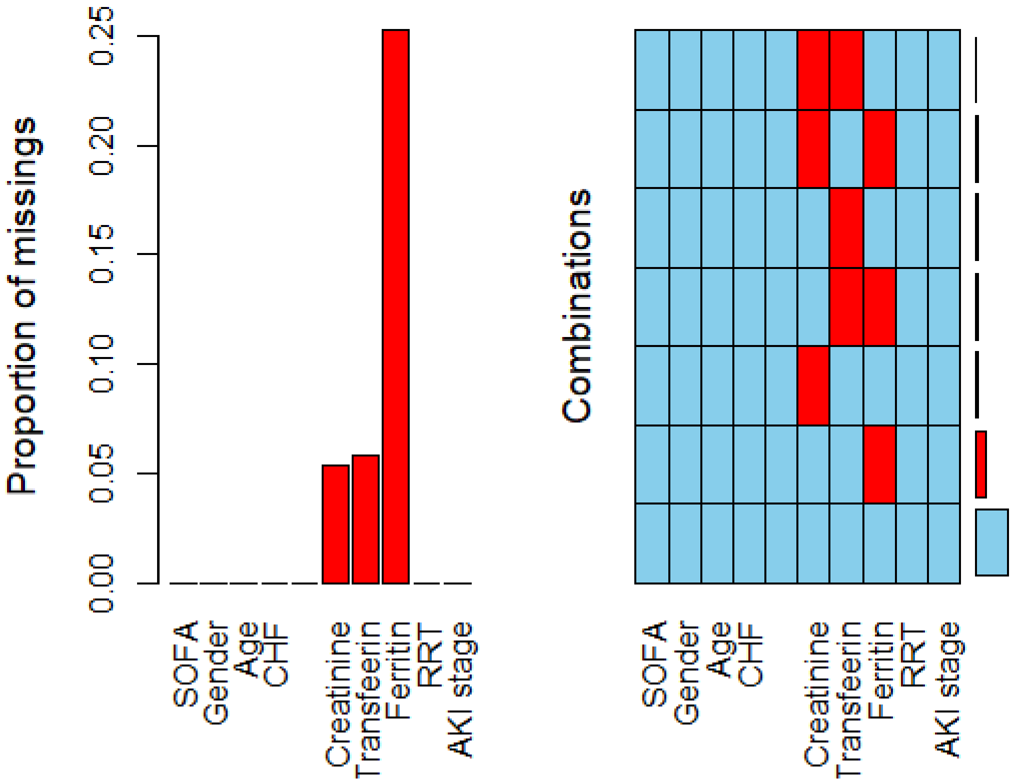

Supplement: Supplementary file 1 — Additional file 1: Figure S1. Pattern of missing data in variables of interest. [file 12882_2020_1965_MOESM1_ESM.pdf]
