# Supplementary material for: Elevated serum iron level is a predictor of prognosis in ICU patients with acute kidney injury
Source: BMC Nephrol. 2020 Jul 25;21:303. doi: 10.1186/s12882-020-01965-9 (PMC7382811; doi:10.1186/s12882-020-01965-9)
Supplement: Supplementary file 3 — Additional file 3: Table S1. Baseline characteristics of the original study cohort. Values are number (%) and mean (SD). SOFA Score, Sequential Organ Failure Assessment score; RRT, Renal replacement therapy; SD, standard deviation. Table S2 Baseline characteristics of the patients in two groups after interpolation. Values are number (%) and mean (SD). SOFA Score, Sequential Organ Failure. Assessment score; RRT, Renal replacement therapy; SD, standard deviation. Table S3. Cox proportional hazards models exploring the relationship between serum iron levels and 28-day mortality. Table S4. Cox proportional hazards models exploring the relationship between serum iron levels and 90-day mortality. [file 12882_2020_1965_MOESM3_ESM.pdf]

Title： Elevated serum iron level is a predictor of prognosis in ICU patients with acute kidney injury

Jie Shu[1], Yufeng HU[1], Xueshu Yu[1], Jiaxiu Chen[1], Wenwei Xu[1], Jingye Pan[1,*]

Affiliations

[1] Department of Intensive Care Unit, Wenzhou Medical University, Wenzhou, 325000, Zhejiang, People's Republic of China

[*] **Corresponding author:** Jing-Ye Pan, The First Affiliated Hospital of Wenzhou Medical University, Wenzhou, Zhejiang Province, 325000, China. E-mail: wmupanjingye@126.com.

Table S1. Baseline characteristics of the original study cohort. Values are presented as number (%) and mean (SD). SOFA Score, Sequential Organ Failure Assessment score; RRT, Renal replacement therapy; SD, standard deviation.

Characteristics	Values
**Total**	483
**Sex, n(%)**	
Female	237 (49.1)
Male	246 (50.9)
**Age, n(%)(years)**	
16-59	196 (40.6)
⩾60	287 (59.4)
**Comorbidity, n(%)**	
Congestive heart failure, n(%)	198 (41.0)
Hypertension, n(%)	255 (52.8)
**SOFA score, n(%)**	
<2	39 (8.1)
⩾2	444 (91.9)
**Stage of AKI, n(%)**	
Stage 1	114 (23.6)
Stage 2	115 (23.8)
Stage 3	254 (52.6)
**RRT, n(%)**	123 (25.5)
**Iron group, n(%)**	
Low iron group	311 (64.4)
High iron group	172 (35.6)
**Laboratory measurements**	
Creatine, mean (SD)(mg/dl)	2.20 (1.61)
Transferrin, mean (SD)(mg/dl)	160.95 (59.44)
Ferritin, mean (SD)(ng/ml)	439.02 (417.24)
**28-day survival status, n(%)**	
Non-death	334 (69.2)
Death	149 (30.8)
**28-day survival times, mean(SD)**	22.34 (9.52)
**90-day survival status, n(%)**	
non-death	292 (60.5)
death	191 (39.5)
**90-day survival times, mean (SD)**	61.76 (37.19)

Table S2. Baseline characteristics of the patients in two groups after interpolation. Values are presented as number (%) and mean (SD). SOFA Score,

Sequential Organ Failure Assessment score; RRT, Renal replacement therapy; SD, standard deviation.

Characteristics	Serum iron < 60	Serum iron ≥ 60	P Value
**Total**	311	172	
**Sex, n(%)**			0.024
Female	165 (53.1)	72 (41.9)	
Male	146 (46.9)	100 (58.1)	
**Age, n(%)(years)**			<0.001
16 - 59	100 (32.2)	96 (55.8)	
⩾ 60	211 (67.8)	76 (44.2)	
**Comorbidity, n(%)**			
Congestive heart failure	159 (51.1)	39 (22.7)	<0.001
Hypertension	173 (55.6)	82 (47.7)	0.114
**SOFA score, n(%)**			0.237
< 2	29 (9.3)	10 (5.8)	
⩾ 2	282 (90.7)	162 (94.2)	
**Stage of AKI, n(%)**			0.174
Stage 1	81 (26.0)	33 (19.2)	
Stage 2	75 (24.1)	40 (23.3)	
Stage 3	155 (49.8)	99 (57.6)	
**RRT, n(%)**	67 (21.5)	56 (32.6)	0.011
**Laboratory measurements**			
Creatine, mean (SD)	2.19 (1.51)	2.35 (1.72)	0.303
Transferrin, mean (SD)	162.76 (58.66)	157.54 (57.97)	0.348
Ferritin, mean (SD)	434.78 (379.43)	607.44 (393.24)	<0.001
**28-day survival status, n(%)**			<0.001
Non-death	233 (74.9)	101 (58.7)	
Death	78 (25.1)	71 (41.3)	
**28-day survival times, mean (SD)**	23.49 (8.56)	20.26 (10.77)	<0.001
**90-day survival status, n(%)**			0.009
Non-death	202 (65.0)	90 (52.3)	
Death	109 (35.0)	82 (47.7)	
**90-day survival times, mean (SD)**	66.22 (35.04)	53.70 (39.63)	<0.001

Table S3. Cox proportional hazards models exploring the relationship between serum iron levels and 28-day mortality.

Group	Multivariate model	
	HR (95%CI)	P value
Original study cohort		
Low iron group	Reference	—

High iron group	1.837(1.166-2.895)	0.009
Interpolated study cohort		
Low iron group	Reference	—
High iron group	1.832(1.305-2.573)	<0.001

Table S4 Cox proportional hazards models exploring the relationship between serum iron levels and 90-day mortality

Group	Multivariate model	
	HR(95%CI)	P value
Original study cohort		
Low iron group	Reference	—
High iron group	1.620（1.073-2.447）	0.022
Interpolated study cohort		
Low iron group	Reference	—
High iron group	1.741(1.285-2.358)	<0.001
